# Influence of Crack Size on Stress Evaluation of Ferromagnetic Low Alloy Steel with Metal Magnetic Memory Technology

**DOI:** 10.3390/ma12244028

**Published:** 2019-12-04

**Authors:** Bin Liu, Peng Fu, Ruifeng Li, Peng He, Shiyun Dong

**Affiliations:** 1 Material Science and Engineering, Jiangsu University of Science and Technology, Zhenjiang 212003, China; baix2415674055@163.com (P.F.); li_ruifeng@just.edu.cn (R.L.); 2 State Key Laboratory of Advanced Welding and Joining, Harbin Institute of Technology, Harbin 150001, China; 3 National Key Laboratory for Remanufacturing, Academy of Armored Forces Engineering, Beijing 100072, China; liubindely@163.com

**Keywords:** non-destructive evaluation, crack effect, metal magnetic memory, magnetic intensity gradient, stress

## Abstract

Based on the magneto-mechanical effect, the influence of crack size on stress evaluated with metal magnetic memory (MMM) technology was discussed in this paper. Based on equivalent theory, the regular rectangular grooves, with different widths and depths, were precut in the surface of an experimental sample for simulating surface crack, and a three dimensional electrically controlled displacement system was used to collect the *H*_p_(*y*) signal of the sample under different stresses, and the fracture morphology was observed by using scanning electron microscopy (SEM). The results show that the influence of detection line on *H*_p_(*y*) signal can be ignored; as stress increases, the *H*_p_(*y*) signal turns counterclockwise around zero-crossing point and its mutation, corresponding to the location of groove, becomes distinct gradually. When groove depth is constant, the magnetic intensity gradient changes in the form of quadratic polynomial as groove width increases, and when the groove width is the same, the magnetic intensity gradient is a linear function of groove depth. When stress reaches the yield strength of the material, the magnetic intensity gradient decreases gradually as stress increases further, and the orientation of magnetic domain is seen as the main reason for that result. At last, the experimental results are discussed based on the piezomagnetic effect and leakage magnetic field theory of finite depth slit model, and the change of magnetic domain orientation is considered to be the main reason.

## 1. Introduction

The safety and reliability of equipment in the working environment has attracted the attention of scholars. However, there is no absolute method for evaluating reliability, so finding a feasible method appears to be particularly urgent. To solve that problem, lots of research has been designed and carried out, and it indicates that stress could be seen as a parameter to characterize reliability, so the method of stress evaluation was studied. In general, the method of stress evaluation can be divided into two categories, including non-destructive and destructive methods [1,2,3,4,5,6,7]. Because of non-destruction, low cost, high efficiency and other merits, the metal magnetic memory (MMM) technology, which is one kind of magnetic flux leakage method, becomes a commonly used method for stress evaluation of ferromagnetic material.

Based on the magnetic memory effect of ferromagnetic material in the geomagnetic field, the MMM technology was firstly proposed by a Russian scholar [8,9,10,11], and it stated clearly that the normal component of MMM signal—*H*_p_(*y*) signal, which was defined in the geomagnetic field, could be employed to evaluate stress. Up to now, lots of research results have been found, however lots of influential factors are found in the application of stress evaluation with this method, and its mechanism is still not elaborated clearly. Thus, lots of experimental and theoretical studies were carried out. For example, the change in the orientation of magnetic domain was explained as the reason for evaluating stress with this technology [12,13,14]. The results of stress effect on *H*_p_(*y*) signal and its influence mechanism were both clarified qualitatively [15]. The grain size effect on *H*_p_(*y*) signal for stress evaluation of low carbon steel was analyzed, and the relation of magnetic intensity gradient and grain size was also obtained [16]. As fatigue cycle numbers increased, the change in the maximal value of surface magnetic flux density of 304 stainless steel was discussed, and the phase transformation was seen as the main reason [17]. Besides that, the fatigue stress of ferromagnetic material was also evaluated, and the relation of fatigue cycle and *H*_p_(*y*) signal was discussed [18,19,20,21,22]. While it should be noted that defects exist inevitably in material, its effect on stress evaluation is discussed rarely, so designing the method for discussing its influence on stress evaluation and explain its results clearly are very important. As a typical defect, the influence of crack on stress evaluated with MMM technology was studied, and its influence degree affected by the crack size was explained in this study. To solve the problem, the equivalent theory was employed, and the regular rectangular groove is used to simulate cracks. After that, the *H*_p_(*y*) signals were collected with the uniaxial static tensile experiment, and then the parameter of *H*_p_(*y*) signal for characterizing stress was determined. At last, the relations between the characteristic parameter of *H*_p_(*y*) signal and crack size was obtained.

## 2. Experimental Material and Methods

### 2.1. Experimental Material

In this study, the experimental material is 12CrMoV, and its mechanical properties are tested and shown in Table 1.

**Figure 1:**
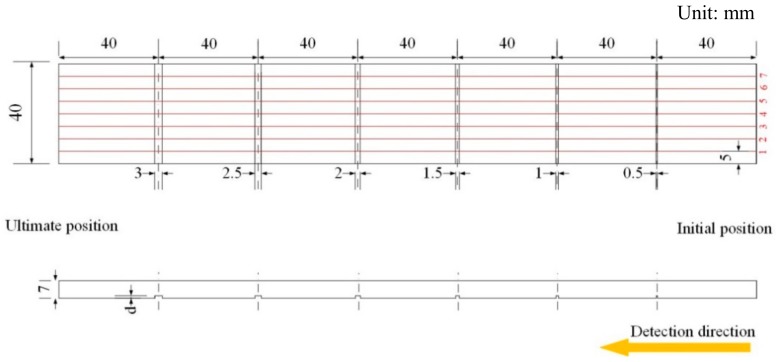
The sketch map of the experimental sample with regular rectangular groove.

In order to discuss and determine the crack effect on stress evaluation, the regular rectangular grooves with different depths and widths were precut in the surface of 12CrMoV by using wire cutting technology, and its sketch map was shown in Figure 1. For experimental requirements, a sample with the size of 280 mm × 40 mm × 7 mm was prepared. In the same sample, the groove depth, the symbol of which is *d* as shown in Figure 1, is the same, and the widths are 0.5 mm, 1.0 mm, 1.5 mm, 2.0 mm, 2.5 mm and 3.0 mm, respectively. For different samples, the groove depths are 0.5 mm, 1.0 mm, 1.5 mm, 2.0 mm, 2.5 mm and 3.0 mm, respectively. To avoid the influence of surface roughness on *H*_p_(*y*) signal, the surfaces of all samples were rubbed, and its surface roughness was Ra1.2. To avoid the deformation influence of material, the spacing between adjacent grooves is determined as 40 mm, and seven detection lines, the spacing between which is 5.0 mm, are drawn on the surface of sample as shown in Figure 1. To eliminate the initial *H*_p_(*y*) signal effect caused by machining, all samples were annealed at 860 °C in the vacuum heat treatment furnace, the vacuity of which was 8 × 10^−4^ Pa. Afterwards, the temperature was reduced to 200 °C and the samples were taken out from the vacuum heat treatment furnace.

### 2.2. Experimental System

**Figure 2:**
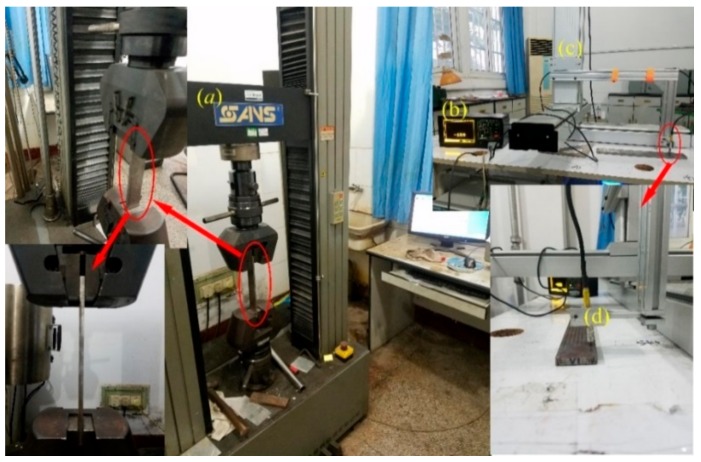
Metal magnetic memory detection system for stress evaluation. (**a**) CMT2502 testing machine. (**b**) EMS-2003. (**c**) Three-dimensional electrically controlled displacement system. (**d**) Two-channel pen sensor probe.

The MMM detection system, shown in Figure 2, consists of three parts. The metal magnetic memory detector—EMS-2003, shown in Figure 2b, is used to store *H*_p_(*y*) signal. The three-dimensional electrically controlled displacement system, shown in Figure 2c, is used to move the two-channel pen sensor probe with a constant scanning speed and lift-off. The displacement system is made of aluminum alloy, so the influence of material magnetism on *H*_p_(*y*) signal can be ignored. The two-channel pen sensor probe, shown in Figure 2d, is used to collect *H*_p_(*y*) signal. For matching the sampling rate of EMS-2003 and the displacement system, the scanning speed of the sensor probe is optimized, and 40 mm/s is optimal. Before collecting *H*_p_(*y*) signal, the samples are firstly placed horizontally on the platform of the displacement system, and then the *H*_p_(*y*) signal is collected along detection lines from initial position to ultimate position, as shown in Figure 1. The static tensile test is done by using a CMT2502 testing machine, shown in Figure 2a, the dynamic load error of which is ±1.0%, meeting the requirements of the experiment.

**Figure 3:**
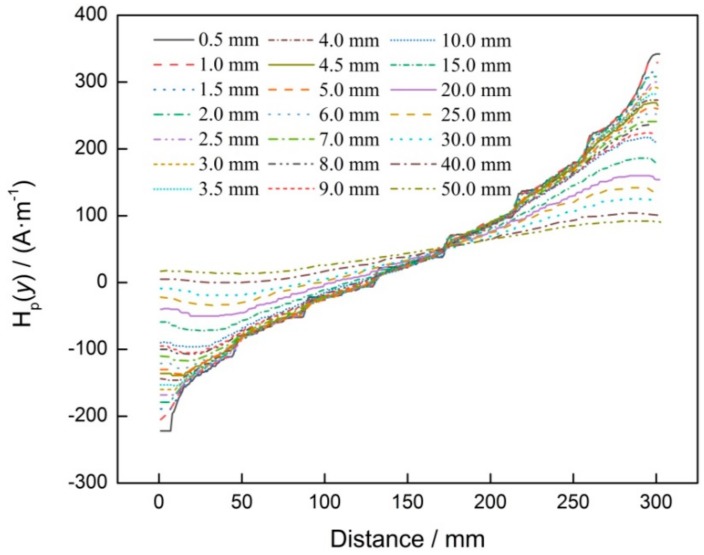
*H*_p_(*y*) signal corresponding to different lift-offs of sensor probe.

Lots of experiments pointed out that lift-off of the sensor probe is an important factor for the amplitude of the *H*_p_(*y*) signal, so the best optimal lift-off is discussed in this study. For the experiment, the sample with the same width and different depths of crack was prepared by using the same technology, which was employed in Figure 1. Increasing the lift-off gradually, the *H*_p_(*y*) signals were collected and shown in Figure 3. It can be seen that when lift-off is lower than 5.0 mm, the mutation of *H*_p_(*y*) signal, at the location of the groove, is clear, and the amplitude of the *H*_p_(*y*) signal is high. As lift-off increased, that mutation becomes smaller and smaller, and the amplitude of the *H*_p_(*y*) signal also becomes lower. After lift-off reaches 15.0 mm, and as lift-off increases further, that mutation disappears and that amplitude becomes lower. With comprehensive consideration, 1.0 mm is seen as the best optimal lift-off of sensor probe in this study. 

## 3. Results and Discussion

### 3.1. Experimental Results

**Figure 4:**
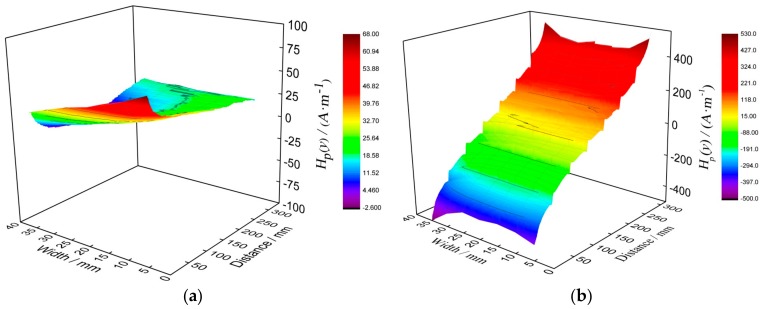

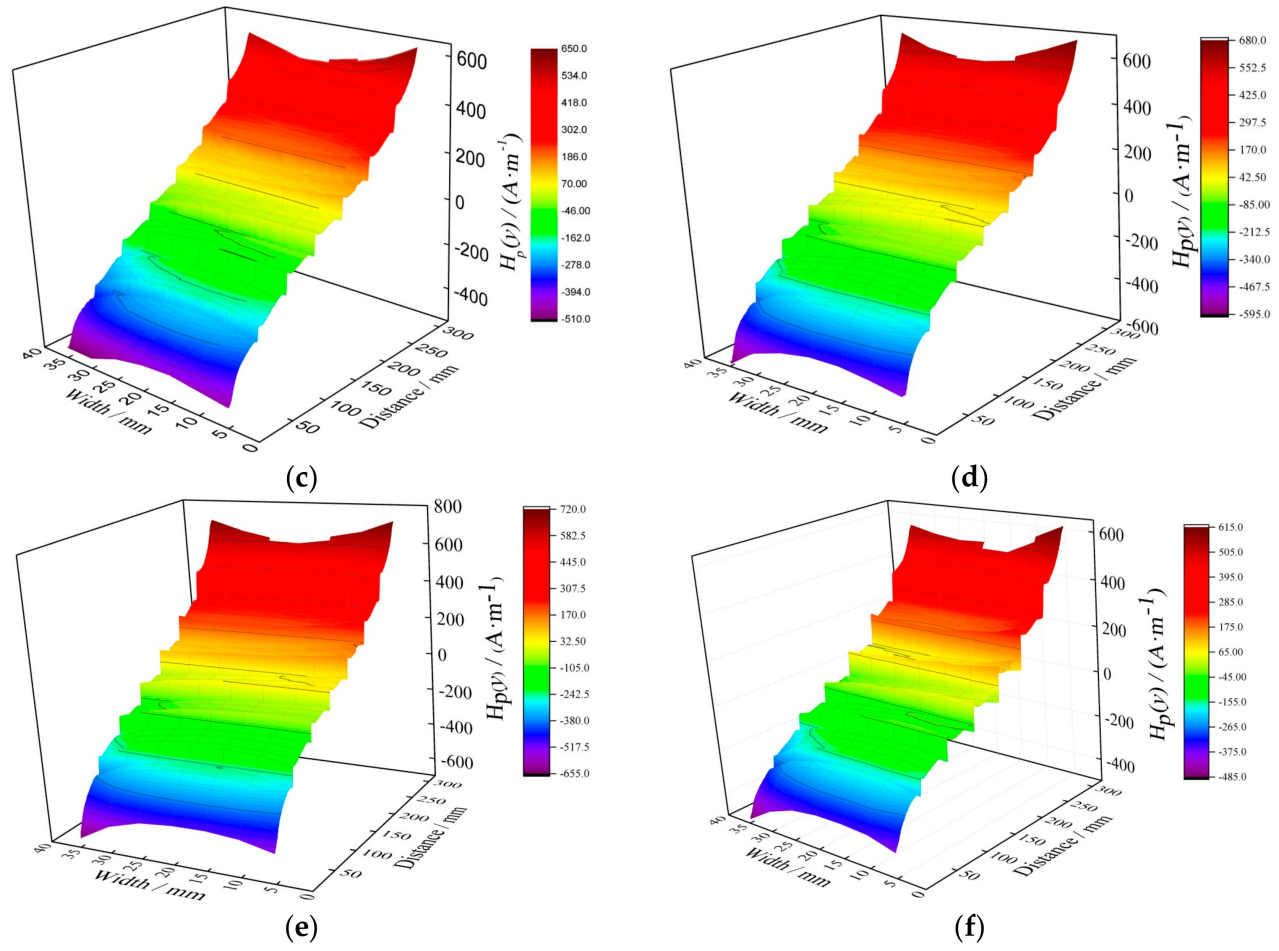
*H*_p_(*y*) signals under different tensile loads. (**a**) 0 MPa, (**b**) 75 MPa, (**c**) 150 MPa, (**d**) 225 MPa, (**e**) 337.5 MPa, (**f**) 375 MPa.

To discuss the crack size effect on stress evaluation, the *H*_p_(*y*) signal, corresponding to different stresses, should be collected in advance with the tensile test, so the preload was determined based on the mechanical properties of material, as shown in Table 1. In this study, the load interval is 6 kN, and the loading rate is 0.5 kN/s. When the preload was reached, the load was held about 120 s, and then the sample was taken down from the CMT2502 testing machine. After that, the sample was placed on the platform, and the *H*_p_(*y*) signals were sequentially collected along detection lines. Comparing the *H*_p_(*y*) signals, it can be seen that although the sizes of cracks are different, the change regulation of the *H*_p_(*y*) signal is very similar as stress changes. Therefore, when the groove depth is 3.0 mm, the *H*_p_(*y*) signals of the sample along seven detection lines, corresponding to different stresses, are shown in Figure 4.

**Figure 5:**
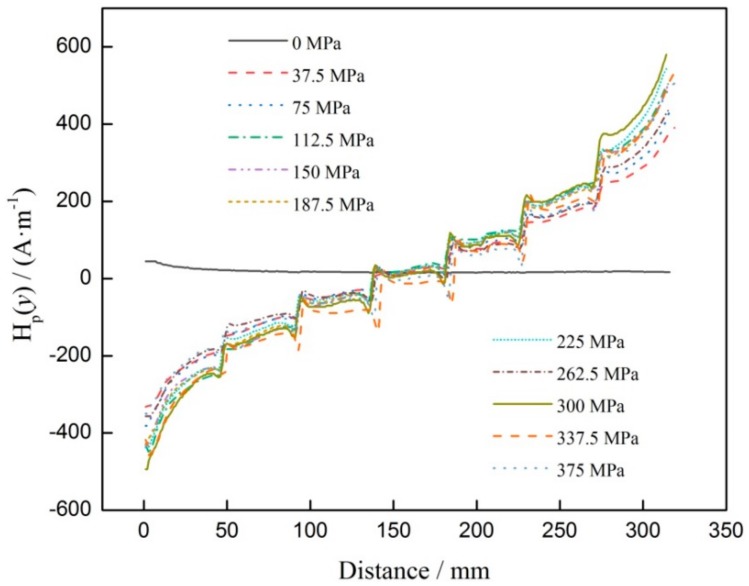
*H*_p_(*y*) signals corresponding to different stresses.

From Figure 4a, it can be seen that when the stress is 0 MPa, the distribution of *H*_p_(*y*) signal is irregular, and its amplitude is in the range of −2.6~67.8 (A·m^−1^). For the result, it can be known that the amplitude is in the range of intensity of the geomagnetic field, thus the initial *H*_p_(*y*) signal effect on stress evaluation can be ignored. From Figure 4b–e, it can be seen that as stress increases, the amplitude of the whole *H*_p_(*y*) signal increases gradually, and the *H*_p_(*y*) signal mutation, corresponding to groove, also becomes more obvious gradually. Compared with the amplitude of *H*_p_(*y*) signal in Figure 4e, it can be seen that the amplitude in Figure 4f decreases as stress increases. Comparing *H*_p_(*y*) signals in different detection lines, it can be seen that when the stress is the same, the *H*_p_(*y*) signals and its changes are basically the same. Based on that result, the *H*_p_(*y*) signals along the middle line are analyzed and shown in Figure 5.

Figure 5 shows that when stress is 0 MPa, the *H*_p_(*y*) signal appears nearly as a horizontal line. As stress increases, the *H*_p_(*y*) signals present linear distribution, then turn anticlockwise and approximately intersect at one point, which is defined as zero crossing point in this paper. For the mutation of *H*_p_(*y*) signal, corresponding to groove, it can be seen that when the groove size is different, the mutation degree also changes, so it can be used to evaluate the stress. Therefore, the mutation of *H*_p_(*y*) signal, corresponding to groove, was extracted and defined as magnetic intensity gradient *K*, which was calculated with Equation (1).

(1) $$K=\frac{H_{p}{\left(y\right)}_{\max}-H_{p}{\left(y\right)}_{\min}}{\Delta l}$$

where *H*_p_(*y*)_max_ and *H*_p_(*y*)_min_ are the maximal and minimal values of *H*_p_(*y*) signal, Δ*l* is the spacing between the location of *H*_p_(*y*)_max_ and *H*_p_(*y*)_min_.

For different crack sizes, the *K* of *H*_p_(*y*) signal was calculated based on Equation (1). It can be seen that when the depth of groove is the same, the relationship between *K* and groove width is very similar. For that reason, not all the experimental results were shown in this paper, and only when the groove depth is 3.0 mm, the relation of *K* and groove width was shown in this paper.

**Figure 6:**
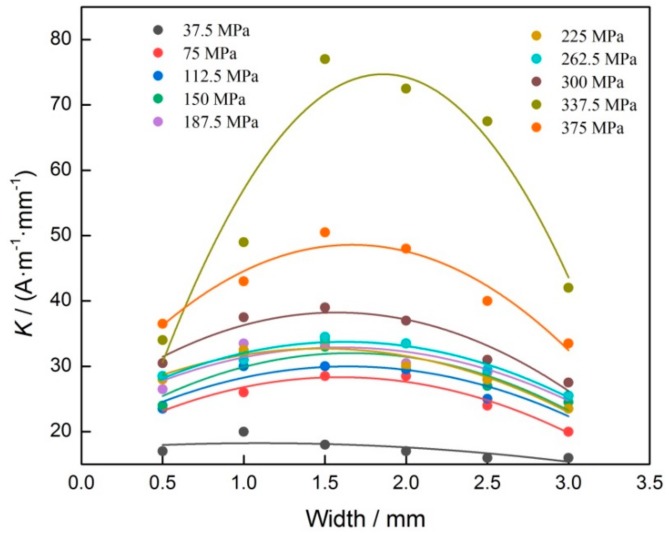
Relation of *K* and width of groove.

When the stresses are different, the curves of *K* and groove width were shown in Figure 6. It can be seen that as groove width increases, the change regulation of *K* is very similar, and it presents in the form of parabola. When stress is 337.5 MPa, which is the yield strength of material, the *K* reaches the maximal value. Based on that, the value of *K* and groove width is fitted with quadratic polynomial function.

**Figure 7:**
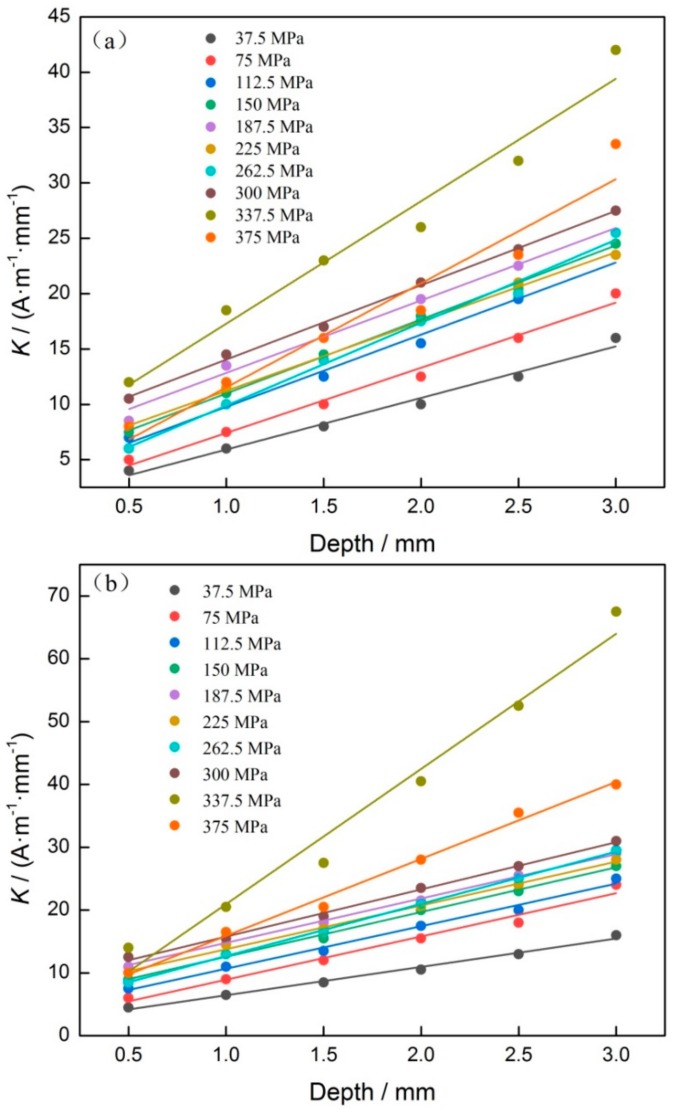
Relationship of *K* and groove depth. (**a**) 2.0 mm, (**b**) 3.0 mm.

Similarly, for discussing the depth effect on stress evaluation, the relation of groove depth and *K* was analyzed. For that purpose, the *H*_p_(*y*) signals, corresponding to the same widths and different stresses, were collected, and its *K* was also calculated. Comparing experimental results, it can be seen that although groove depths are different, the change regulation of *K* is very similar as stress increases gradually. In view of that, when groove widths were 2.0 mm and 3.0 mm, which were the common widths, the results of *K* and groove depth were shown in Figure 7.

**Figure 8:**
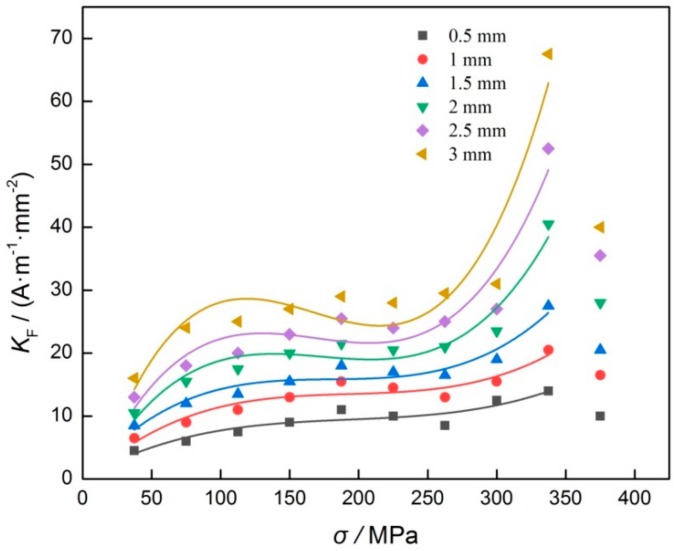
Result of *K_F_* and tensile stress.

As shown in Figure 7, it can be seen that the relationship of *K* and groove depth is nearly linear, and the slope between *K* and groove depth increases gradually as stress increases. When stress reaches 337.5 MPa, the slope reaches the maximal value, and then becomes smaller as stress increases further. To discuss the influence of stress on the value of *K*, the result of *K* and stress was fitted with linear function. In this study, the fitting coefficient was defined as *K_F_*, so the relationship between *K_F_* and stress was determined and shown in Figure 8.

As shown in Figure 8, it can be seen that although the groove widths are different, the change regulation of *K_F_* is basically the same as stress increases. In detail, when the stress is 337.5 MPa, the *K_F_* corresponds to the maximal value, and it decreases obviously as stress increases further. For the same stress, the *K_F_* increases gradually as the groove width increases.

### 3.2. Discussion and Analysis

**Figure 9:**
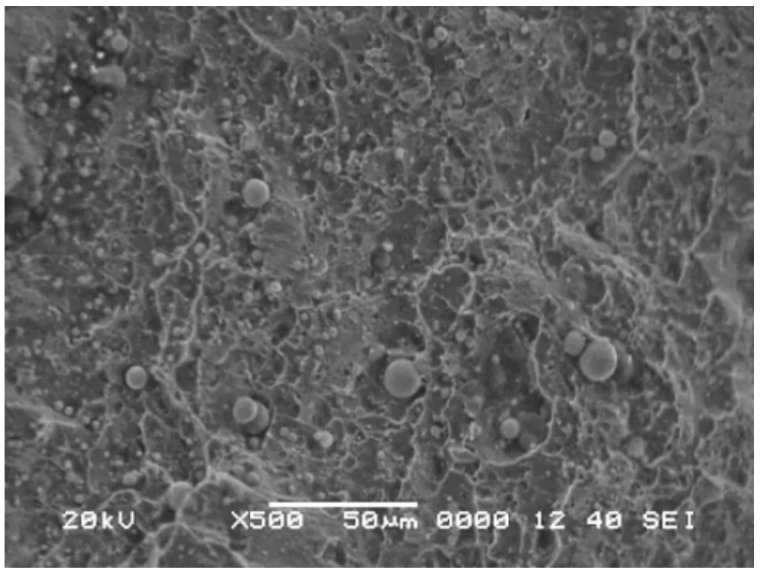
The fracture morphology of the experimental sample.

In order to explain the experimental results, the magneto-mechanical effect and the finite depth slit leakage magnetic field theory are employed. It is generally known that after demagnetization of magnetic material, the orientation of magnetic domain is random, so when stress is 0 MPa, the amplitude of *H*_p_(*y*) signal is very low in experimental results. When the stress is lower than the yield strength of material, as stress increases, the orientation of the magnetic domain becomes orderly gradually. For that process, the change in orientation of the magnetic domain is described as three stages, including magnetic domain movement, magnetic domain merger and magnetic domain rotation; after that, the orientation is parallel to the stress direction. It indicates that as stress increases, the magnetic characteristic of material becomes stronger, so the amplitude of *H*_p_(*y*) signal becomes higher gradually as shown in Figure 4. When the stress reaches yield strength, as the stress increases further, lots of dislocations are generated and gathered together in material, and an internal stress field is formed simultaneously. Because there is a pinning effect of dislocation on the magnetic domain, which has been reported by many studies, the orientation change in the magnetic domain is restricted as stress increases. As we all know, the magnetization degree of material largely depends on the order degree of magnetic domain orientation, thus the magnetization degree is decreased as stress increases in the plastic deformation stage. Thist means that the amplitude of *H*_p_(*y*) signal decreases, as shown in Figure 4f. However, there were some studies showing that when the material was in the state of plastic deformation, the magnetization degree still increased gradually as stress increased, because that pinning effect could be broken. However, some other scholars had different opinions, and they thought that for the material with good plastic deformation capability, that pinning effect was hardly broken [23]. For the material in this study, its plastic deformation capability is very good, so a larger number of dislocations are generated in the plastic deformation stage, and the pinning effect of dislocation cannot be broken. To verify the theoretical analysis, the fracture morphology of experimental material was observed and shown in Figure 9.

From Figure 9, it can be seen that many dimples are generated, which can be seen in the material fracture. The fracture theory of metal states clearly that more dimples represent better deformation performance, thus the experimental result agrees well with the theoretical analysis.

**Figure 10:**
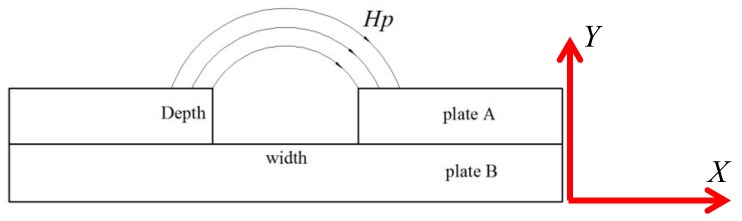
The leakage magnetic field model of finite depth slit.

To explain the change in *H*_p_(*y*) signal, corresponding to grooves, the leakage magnetic field model of finite depth slit, which was built by Förster [24], was employed, and it was shown in Figure 10.

As the definition of the coordinate (*X*,*Y*), shown in Figure 10, the leakage magnetic field intensity can be written as:

(2) $$H_{y}=\frac{(l+W)\mu H_{0}W}{\pi (l+\mu W)}[\frac{x}{x^{2}+y^{2}}-\frac{x}{x^{2}+{\left(y+D\right)}^{2}}]$$

where *H_y_* is the normal component of leakage magnetic field, *x* is the horizontal distance between testing point and the center of slit, *y* is the vertical distance between testing point and the center of slit, *W* and *D* are the width and depth of the slit, *H*_0_ is internal magnetic field intensity of material, *l* is the length of sample, and *μ* is the magnetic permeability.

Generally speaking, the *H*_0_, which is determined by the earth’s magnetic field and the magnetic field induced by stress, can be written as:

(3) $$H_{0}=H_{E}+\frac{H_{E}M_{S}(\alpha \mu_{0}+3\gamma \sigma )}{3\alpha \mu_{0}-M_{S}(\alpha \mu_{0}+3\gamma \sigma )}$$

where *H*_E_ is the earth’s magnetic field intensity, *M*_S_ is the saturation magnetization of sample, *α* is the parameter of molecular field determined by material, *μ*_0_ is the vacuum permeability, and *γ* is determined by the magnetostrictive coefficient.

Taking Equation (3) into Equation (2), the *H_y_* can be written as:

(4) $$H_{y}=[H_{E}+\frac{H_{E}M_{S}(\alpha \mu_{0}+3\gamma \sigma )}{3\alpha \mu_{0}-M_{S}(\alpha \mu_{0}+3\gamma \sigma )}]\frac{(l+W)\mu W}{\pi (l+\mu W)}[\frac{x}{x^{2}+y^{2}}-\frac{x}{x^{2}+{\left(y+D\right)}^{2}}]$$

Then, taking Equation (4) into Equation (1), the magnetic intensity gradient *K*, which is defined in Equation (1), can be expressed as:

(5) $$\begin{array}{c} K=\frac{H_{y1}-H_{y2}}{x_{1}-x_{2}}\\ =[H_{E}+\frac{H_{E}M_{S}(\alpha \mu_{0}+3\gamma \sigma )}{3\alpha \mu_{0}-M_{S}(\alpha \mu_{0}+3\gamma \sigma )}]\cdot \frac{(l+W)\mu W}{\pi (l+\mu W)}\cdot [\frac{x_{1}(y+D)}{\left(x_{1}^{2}+y^{2})(x_{1}^{2}+{\left(y+D\right)}^{2}\right)}-\frac{x_{2}(y+D)}{\left(x_{2}^{2}+y^{2})(x_{2}^{2}+{\left(y+D\right)}^{2}\right)}]\cdot \frac{1}{x_{1}-x_{2}} \end{array}$$

To discuss the relationship of *K* and groove width, the partial derivation of *K* respect to *W* is calculated and reduced as:

(6) $$K^{\prime }=[H_{E}+\frac{H_{E}M_{S}(\alpha \mu_{0}+3\gamma \sigma )}{3\alpha \mu_{0}-M_{S}(\alpha \mu_{0}+3\gamma \sigma )}]\cdot \frac{1}{x_{1}-x_{2}}\cdot \frac{\mu W^{2}+2WL+L^{2}}{{\left(L+\mu W\right)}^{2}}\cdot [\frac{x_{1}(y+D)}{\left(x_{1}^{2}+y^{2})[x_{1}^{2}+{\left(y+D\right)}^{2}\right]}-\frac{x_{2}(y+D)}{\left(x_{2}^{2}+y^{2})[x_{2}^{2}+{\left(y+D\right)}^{2}\right]}]$$

where *K*′ is defined as partial derivation of *K* respect to *W*.

Comparing the value of *L* and *μ*, it can be known that the value of *L* >> *μ* can be accepted, so Equation (6) is simplified to:

(7) $$K^{\prime }=[H_{E}+\frac{H_{E}M_{S}(\alpha \mu_{0}+3\gamma \sigma )}{3\alpha \mu_{0}-M_{S}(\alpha \mu_{0}+3\gamma \sigma )}]\cdot \frac{1}{x_{1}-x_{2}}\cdot \frac{2W+L}{L}\cdot [\frac{x_{1}(y+D)}{\left(x_{1}^{2}+y^{2})[x_{1}^{2}+{\left(y+D\right)}^{2}\right]}-\frac{x_{2}(y+D)}{\left(x_{2}^{2}+y^{2})[x_{2}^{2}+{\left(y+D\right)}^{2}\right]}]$$

In this study, the groove depth is constant in the same one sample, meaning that the value of *D* is a constant, so the relation of *K*′ and *W* is linear in Equation (7). Based on the derivative theory, it can be known that *K* is a quadratic polynomial function of *D*, so it agrees well with the experimental result, as shown in Figure 6.

Similarly, the relation of *K* and *D* is discussed. The partial derivation of *K* respect to *D* is calculated, so the Equation (5) is reduced to:

(8) $$\begin{array}{c} K\prime \prime =[H_{E}+\frac{H_{E}M_{S}(\alpha \mu_{0}+3\gamma \sigma )}{3\alpha \mu_{0}-M_{S}(\alpha \mu_{0}+3\gamma \sigma )}]\cdot \frac{1}{x_{1}-x_{2}}\cdot \frac{\mu (L+W)W}{(L+\mu W)\pi }\cdot \\ \left[\frac{1}{\left(x_{1}^{2}+y^{2})[x_{1}^{2}+{\left(y+D\right)}^{2}\right]}\cdot [\frac{x_{1}^{3}}{x_{1}^{2}+{\left(y+D\right)}^{2}]}-x_{1}]-\frac{1}{\left(x_{2}^{2}+y^{2})[x_{2}^{2}+{\left(y+D\right)}^{2}\right]}\cdot [\frac{x_{2}^{3}}{x_{2}^{2}+{\left(y+D\right)}^{2}]}-x_{2}]\right] \end{array}$$

where *K*″ is defined as the partial derivation of *K* respect to *D*. 

Simplifying Equation (8), it can be written as:

(9) $$\begin{array}{c} K\prime \prime =[H_{E}+\frac{H_{E}M_{S}(\alpha \mu_{0}+3\gamma \sigma )}{3\alpha \mu_{0}-M_{S}(\alpha \mu_{0}+3\gamma \sigma )}]\cdot \frac{1}{x_{1}-x_{2}}\cdot \frac{\mu (L+W)W}{(L+\mu W)\pi }\cdot \\ \left[\frac{x_{1}^{3}}{(x_{1}^{2}+y^{2}){\left[x_{1}^{2}+{\left(y+D\right)}^{2}\right]}^{2}}-\frac{x_{2}^{3}}{(x_{1}^{2}+y^{2}){\left[x_{2}^{2}+{\left(y+D\right)}^{2}\right]}^{2}}-\frac{x_{1}}{\left(x_{1}^{2}+y^{2})[x_{1}^{2}+{\left(y+D\right)}^{2}\right]}+\frac{x_{2}}{\left(x_{2}^{2}+y^{2})[x_{2}^{2}+{\left(y+D\right)}^{2}\right]}\right] \end{array}$$

Because the value of *x* is far bigger than the value of *D* in this study, the influence of change in *D* on *K*″ can be ignored. It means that the relationship of *K* and *D* can be seen as linear, so it agrees well with results as shown in Figure 7. In a word, the experimental result agrees well with theoretical discussion.

## 4. Conclusions

The influence of crack size on stress evaluation of low alloy steel with MMM technology was discussed in this study. It can be concluded that:

1. The *K* of *H*_p_(*y*) signal is a quadratic polynomial function of groove width, and the value of *K* increases gradually as stress increases. When the stress reaches yield strength of the material, the value of *K* also reaches maximum.
2. For different tensile stresses, the *K* is a linear function of groove depth, and its linear slope increases as stress increases gradually. When the stress reaches yield strength of the material, the linear slope reaches the maximal value.
3. For different groove widths, the relationship of *K_F_* and stress is very similar, and it appears nonlinearly.

## Figures and Tables

**Table 1:** Mechanical property of the experimental material.

Mechanical Property	σ_s_/MPa	σ_b_/MPa	δ_5_/%	φ/%
12CrMoV	337.5	547	22	50
